# Efficiency of CIDR-Based Protocols Including GnRH Instead of eCG for Estrus Synchronization in Sheep

**DOI:** 10.3390/ani9040146

**Published:** 2019-04-03

**Authors:** Paula Martinez-Ros, Antonio Gonzalez-Bulnes

**Affiliations:** 1 Dpto. Produccion y Sanidad Animal, Facultad de Veterinaria, Universidad Cardenal Herrera-CEU, CEU Universities, C/Tirant lo Blanc, 7.46115 Alfara del Patriarca, Valencia, Spain; 2 Dpto. de Reproduccion Animal, INIA, Avda. Puerta de Hierro s/n., 28040 Madrid, Spain; bulnes@inia.es; 3 Dpto. de Toxicologia y Farmacologia, Facultad de Veterinaria, UCM, Ciudad Universitaria s/n., 28040 Madrid, Spain

**Keywords:** CIDR, eCG, estrus synchronization, fertility, GnRH, ovulation, sheep

## Abstract

**Simple Summary:**

This study examines the preovulatory and ovulatory events (in terms of the timing of onset of estrus behavior, preovulatory LH surge, and ovulation) and the yields obtained (in terms of ovulation rate, progesterone secretion, and fertility) after insertion of controlled internal drug release (CIDR) devices for 5 days and treatment with equine chorionic gonadotrophin (eCG) or gonadotrophin-releasing hormone (GnRH).

**Abstract:**

The present study examined, for meat sheep (Segureña breed; 2–5-years old, mean body score of 3.5 ± 0.5), the timings of onset of estrus behavior, preovulatory luteinizing hormone (LH) surge and ovulation, and the ovulation rate and fertility obtained after insertion of controlled internal drug release (CIDR) devices for 5 days plus treatment with equine chorionic gonadotrophin (eCG; single dose at CIDR removal, n = 19 ewes) or gonadotrophin-releasing hormone (GnRH, either in a single dose at 56 h after CIDR removal, group CIDR-GnRH, n = 19 ewes; or in one dose at CIDR insertion and another dose 56 h after CIDR removal, group GnRH-CIDR-GnRH, n = 19 ewes). In all the ewes, the appearance of estrus behavior ranged between 84% and 90% and all females showing estrus signs had subsequent preovulatory LH peaks and ovulations. Onset of these events was earlier in the CIDR-eCG group than in the CIDR-GnRH and GnRH-CIDR-GnRH groups (*p* < 0.05). These differences were mainly determined by the onset of estrus behavior, since timing and intervals of LH peak and ovulation were similar among treatments. In fact, the range of ovulations was narrower in the GnRH-CIDR-GnRH group, which suggests better synchronization of follicular growth (*p* < 0.05). In conclusion, protocols with two doses of GnRH offer similar yields to eCG protocols.

## 1. Introduction

Reproductive management of sheep, like in other domestic species, is commonly based on the induction and synchronization of estrus and ovulation, either in reproductive or nonreproductive season and either for natural mating or artificial insemination, by the use of pharmacological treatments [1]. Such treatments, from seminal studies in the 1950s [2], are mostly based on the administration of progesterone or its analogues for mimicking the activity of the corpus luteum. Usually, a single intramuscular dose of equine chorionic gonadotrophin (eCG) is injected at progesterone withdrawal since, from very early studies [3], eCG proved to be effective at inducing estrus and ovulation during seasonal anestrous in order to increase the percentage of twin pregnancies throughout the year and to adjust the interval between ovulation and insemination in protocols for fixed-time artificial insemination (FTAI).

The future use and availability of eCG, despite being an essential component of protocols for induction and synchronization of estrus and ovulation, has been, however, strongly compromised by a highly active animal-rights movement because the hormone is obtained from pregnant mares. Hence, there is a need for alternative protocols without eCG.

We have recently evaluated the usefulness of different short-term (5–7 days) protocols using controlled internal drug release (CIDR) inserts without eCG administration at device removal [4]. Our objective was to prove the hypothesis that such protocols would induce estrus and fertile ovulations without the need for eCG. CIDR insertion causes atresia of any large follicle present in the ovaries and therefore promotes the appearance of new follicles that reach their maximum diameter 5–7 days later [5]. Our results indicated that, without eCG, preovulatory events and fertility after 5 days are better than after 6 or 7 days of CIDR insertion and similar to those obtained by the use of classical treatments with 14 days of CIDR insertion plus eCG at device removal. These findings may indicate that the dominant follicle induced by CIDR insertion would be at the height of its growing phase 5 days later and, in case of CIDR withdrawal, would be able to ovulate without eCG stimulation. However, 1 or 2 days after, it would be in the static or regressing phase and, therefore, its ability to ovulate without eCG stimulation would be compromised. 

The proposed protocol of 5 days of CIDR insertion may be useful for natural mating, but its use in FTAI requires the precise synchronization of ovulations obtained by the use of eCG. In fact, our previous study indicates that fertility under field conditions was similar between treatments based on 14 days of CIDR plus eCG and 5 days of CIDR without eCG but increased around 20% when using 5 days of CIDR plus eCG. These results highlight the benefits of using eCG regardless of the duration of CIDR insertion and encourage the search for an alternative.

A possible option would be other luteinizing hormone (LH)-active hormones, but early studies evaluating the usefulness of gonadotrophin-releasing hormone (GnRH), human chorionic gonadotrophin (hCG) and even a eCG-hCG mixture (PG600) indicated that only GnRH would constitute a potential substitute [6]. In fact, later studies trying to replace eCG with hCG, at least partly if using PG600, showed poorer fertility yields [7,8]. The most promising results have been found when using GnRH to stimulate ovulation [9], although this hormone has been more frequently associated with prostaglandin-based protocols [10,11,12]. It is important to note that GnRH must be applied at least 24–36 h after progestagen removal or luteolysis [13], since its earlier application (e.g., at progestagen removal) causes luteinization of the preovulatory follicle and anovulation [14].

The first hypothesis to be tested in the current work was that GnRH may synchronize ovulations after 5 days of CIDR treatment in a similar way to eCG, giving similar reproductive yields. The second hypothesis was that the administration of GnRH at CIDR insertion would facilitate a more precise synchronization of the follicular wave; such a protocol including a first GnRH dose at CIDR insertion and a second dose concomitantly with artificial insemination would be similar to protocols using a Cosynch protocol [15] supplemented with progesterone [16] and, therefore, would contribute to a more precise synchronization of the ovulatory events, thus improving fertility.

Hence, the objective of the present study was to characterize the preovulatory and ovulatory events (in terms of timing of onset of estrus behavior, preovulatory LH surge, and subsequent ovulation) and the yields obtained (in terms of ovulation rate, progesterone secretion, and fertility) after the insertion of a CIDR device for 5 days combined with either the administration of eCG at sponge removal or the administration of two different schemes of GnRH treatment. These results would have direct implications for evaluating the effectiveness of the proposed practice of using short-term CIDR protocols without eCG.

## 2. Material and Methods

### 2.1. Animals and Experimental Design

The trial was carried out during the breeding season (December) and involved 57 multiparous meat ewes (Segureña breed; 2–5-years old, with a mean body score of 3.5 ± 0.5 on a scale of 1–5), maintained outdoors with access to indoor facilities at the experimental farm of the CEU Cardenal Herrera University in Naquera (Valencia, Spain; latitude 39° N). The experiment was assessed and approved by the University Committee of Ethics in Animal Research (report CEEA17/019) according to the Spanish Policy for Animal Protection (RD53/2013), which meets the European Union Directive 2010/63/UE.

**Figure 1:**
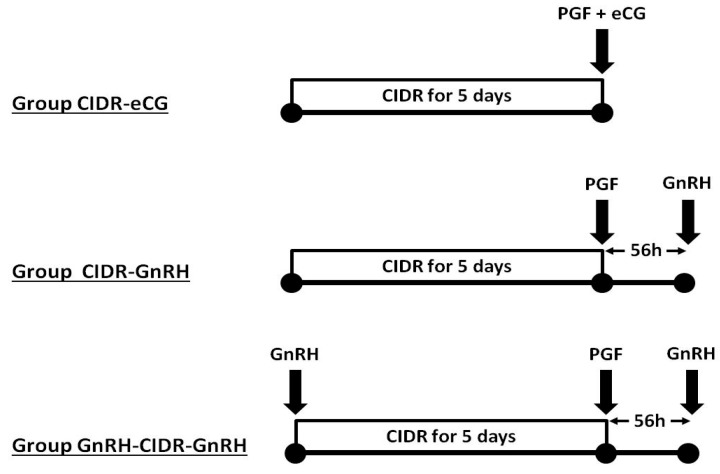
Schematic representation of the three treatment groups compared in the study. All the groups were treated with controlled internal drug release (CIDR) devices for 5 days and a prostaglandin F_2α_ injection at CIDR removal. The CIDR-equine chorionic gonadotrophin (eCG) group was treated with a single intramuscular (i.m.) dose of 400 IU of eCG at CIDR removal, the CIDR-gonadotrophin-releasing hormone (GnRH) group was treated with a single dose of GnRH at 56 h after CIDR removal, and the GnRH-CIDR-GnRH group was treated with two single GnRH doses—one at CIDR insertion and one 56 h after CIDR removal.

Ovarian cyclic activity and ovulation were synchronized in all the animals by the insertion of one intravaginal progesterone-loaded CIDR (CIDR^®^ Ovis, Zoetis, Madrid, Spain) for 5 days and an intramuscular (i.m.) injection of 5 mg of prostaglandin F_2α_ (dinoprost tromethamine, Dinolytic^®^, Zoetis, Madrid, Spain) at CIDR withdrawal, in agreement with previous studies of our group [4], as depicted in Figure 1. The first group of ewes (group CIDR-eCG, n = 19) was treated with a single i.m. dose of 400 IU of eCG at CIDR removal (Foligon^®^, MSD Animal Health, Madrid, Spain), while the other two groups were not treated with eCG. In one of them (group CIDR-GnRH, n = 19), a single dose of 50 µg of GnRH was administered 56 h after CIDR removal (gonadorelin acetate, Acegon^®^, Lab. Syva, Leon, Spain). The third group (group GnRH-CIDR-GnRH, n = 19) received the same treatment as the CIDR-GnRH group but with the addition of a 50-µg GnRH dose at CIDR insertion.

The variables evaluated during the induced follicular phase and the subsequent luteal phase were the percentage and timing of onset of estrus behavior, preovulatory LH surge and ovulation, the number and functionality (in terms of plasma progesterone concentrations) of the induced corpora lutea, and the fertility rate.

### 2.2. Occurrence and Timing of Estrus Behavior Onset

Occurrence and timing of onset of estrus behavior (defined as acceptance of the first mating and time elapsed from CIDR withdrawal) were determined by individual detection of estrus signs by trained rams every 4 h from 20 to 60 h after CIDR withdrawal.

### 2.3. Occurrence and Timing of Preovulatory LH Surge

Occurrence and timing of onset of the preovulatory LH surge (defined as the point before LH concentration increased more than 10% over basal concentrations [17]) were determined by enzimoimmunoassay (LH Detect^®^, INRA, Tours, France) in jugular blood plasma. Plasma samples were collected every 4 h from 32 to 84 h after CIDR removal in heparinized 4-mL vacuum tubes (Vacutainer™ Systems Europe, Meylan, France) and immediately centrifuged at 1500× *g* for 15 min. The plasma was separated and biobanked into polypropylene vials at −80 °C until assayed. The assay had a sensitivity of 0.01 ng/mL and inter- and intra-assay variation coefficients of 7.4% and 8.5%, respectively; variation coefficients were calculated using controls ranging from 0.05 to 40 ng/mL.

### 2.4. Occurrence and Timing of Ovulation

Occurrence and timing of ovulation were determined by transrectal ultrasonography (7.5 MHz; Aloka SSD 500, Aloka Co., Ltd., Tokyo, Japan) by assessing the disappearance of large anechoic structures (i.e., ovulatory follicles) recorded in a previous ultrasonography, as previously described [18,19].

### 2.5. Ovulation Rate and Corpora Lutea Functionality

Ovulation rate was determined by transrectal ultrasonography at Day 10 of the induced estrous cycle. Concomitantly, jugular blood plasma samples, obtained as previously described, were used to determine plasma progesterone concentrations with a direct solid-phase RIA kit (PROG-CTRIA, IBA Molecular, Madrid, Spain). The assay had a sensitivity of 0.05 ng/mL and inter- and intra-assay variation coefficients of 4.5% and 3.5%, respectively; variation coefficients were calculated using controls ranging from 0.5 to 20 ng/mL.

### 2.6. Fertility Rate

The fertility rate, in terms of number of pregnant females with regards to treated and mated ewes, was assessed by transrectal ultrasonography at Day 35 after CIDR withdrawal.

### 2.7. Statistical Analysis

Statistical analysis was performed using SPSS^®^ 22.0 (IBM Corporation, Armonk, NY, USA). The effects of treatment (eCG, single GnRH injection, or double GnRH injection) on the onset of estrus behavior; preovulatory LH surge and ovulation and, afterwards, on ovulation rate; and progesterone secretion were assessed by analyses of variance (ANOVA). Ranges in the timing of these variables were analyzed after comparing the 95% credible interval of the mean with the range. Statistical analysis of occurrence of estrus, LH surge, ovulation, and fertility was performed by a chi-squared test after arcsine transformation of the values. All results are expressed as mean ± SEM and the statistical significance was accepted at *p* < 0.05.

## 3. Results

### 3.1. Occurrence and Timing of Estrus Behavior

**Figure 2:**
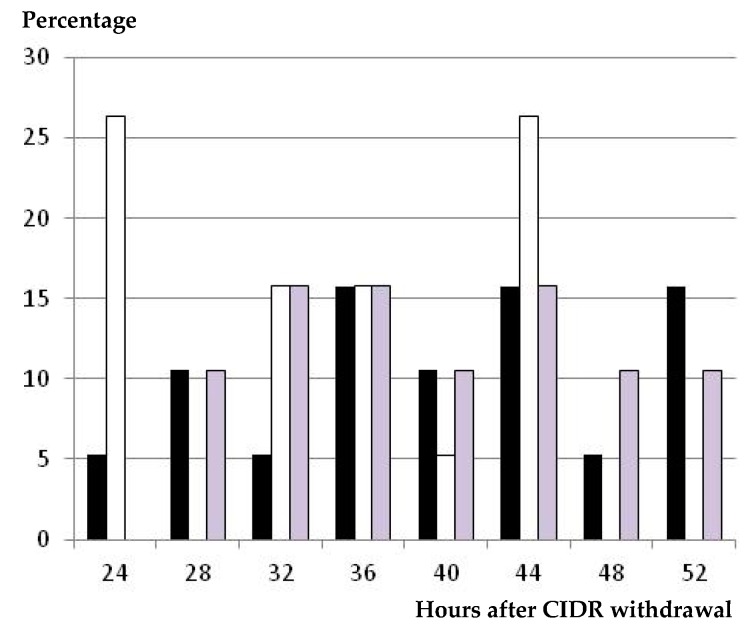
Distribution (percentage of animals) of time of onset of estrus behavior, in hours after CIDR withdrawal, in ewes treated with a single i.m. dose of 400 IU of eCG at CIDR removal (group CIDR-eCG, white bars), with a single dose of GnRH at 56 h after CIDR removal (group CIDR-GnRH, grey bars), and with two GnRH doses—one at CIDR insertion and one 56 h after CIDR removal (group GnRH-CIDR-GnRH, black bars).

The percentage of animals showing estrus behavior after CIDR withdrawal ranged between 84% and 90% in all groups, without significant differences among them (Table 1). There were, on the other hand, significant differences in the timing of estrus onset, and the group treated with eCG had a significantly earlier appearance of estrus signs than the groups treated with GnRH (*p* < 0.05). The range of appearance of estrus behavior was similar for the CIDR-eCG and CIDR-GnRH groups (20 and 24 h, respectively) and longer (36 h; *p* < 0.05) for the GnRH-CIDR-GnRH group (Figure 2).

### 3.2. Occurrence and Timing of Preovulatory LH Surge

**Figure 3:**
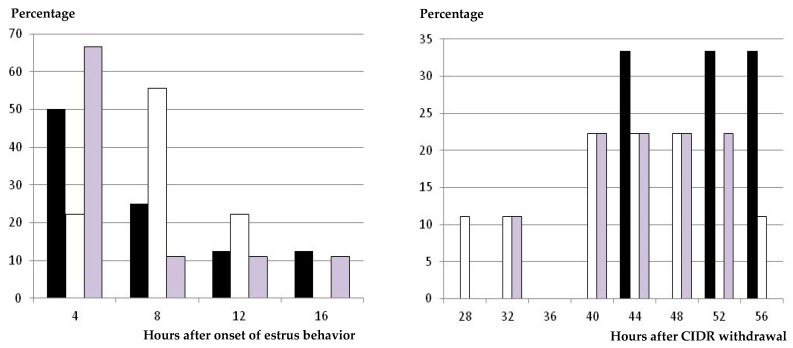
Distribution (percentage of animals) of time of onset of the preovulatory LH surge, in hours after estrus behavior (**left hand**) and after CIDR withdrawal (**right hand**), in ewes treated with a single i.m. dose of 400 IU of eCG at CIDR removal (group CIDR-eCG, white bars), with a single dose of GnRH at 56 h after CIDR removal (group CIDR-GnRH, grey bars), and with two GnRH doses—one at CIDR insertion and one 56 h after CIDR removal (group GnRH-CIDR-GnRH, black bars).

All the animals showing signs of estrus behavior in response to the hormonal treatment, independently of the group, showed a preovulatory LH surge afterwards (Table 1). The timing of the preovulatory surge was earlier in the group treated with eCG than in the GnRH-CIDR-GnRH group (*p* < 0.05), and the CIDR-GnRH group had in-between values. The same was found when analyzing the range of appearance and intervals (Figure 3). These differences were determined by the timing of onset of estrus behavior, since both the mean interval and the range between the onsets of estrus behavior and LH surge were similar between treatments.

### 3.3. Occurrence and Timing of Ovulation

**Figure 4:**
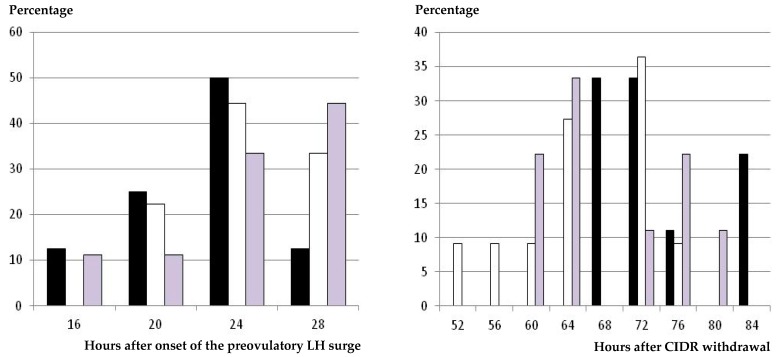
Distribution (percentage of animals) of time of occurrence of ovulation, in hours after onset of the preovulatory LH surge (**left hand**) and after CIDR withdrawal (**right hand**), in ewes treated with a single i.m. dose of 400 IU of eCG at CIDR removal (group CIDR-eCG, white bars), with a single dose of GnRH at 56 h after CIDR removal (group CIDR-GnRH, grey bars), and with two GnRH doses—one at CIDR insertion and one 56 h after CIDR removal (group GnRH-CIDR-GnRH, black bars).

The results on occurrence and timing of ovulation were very similar to the previous data on occurrence and timing of preovulatory LH surge. All the animals showing estrus signs and a preovulatory LH surge afterwards ovulated in response to the hormonal treatment independently of the group (Table 1). The timing of ovulation after CIDR withdrawal was again earlier in the group treated with eCG than in the GnRH-CIDR-GnRH group (*p* < 0.05), and the CIDR-GnRH group again had intermediate values. However, again, these findings were determined by the timing of onset of estrus behavior (Figure 4), since the mean intervals and the range between the occurrences of the preovulatory LH surge and ovulation were similar among treatments. 

Finally, grouping of preovulatory LH surges and ovulations in the GnRH groups caused the range of ovulations after CIDR removal to be narrower in the GnRH-CIDR-GnRH group (16 h) than in the CIDR-GnRH and CIDR-eCG groups (20 and 24 h, respectively).

### 3.4. Ovulation Rate and Corpora Lutea Functionality

There were no significant differences in the mean number of corpora lutea between the groups treated with GnRH (Table 2); however, the ovulation rate was numerically higher in the CIDR-eCG group than in the GnRH-CIDR-GnRH group (*p* = 0.08) and significantly higher in both groups than in the CIDR-GnRH group (*p* < 0.05). Assessment of plasma progesterone did not show significant differences among groups (Table 2).

### 3.5. Fertility Rate

The fertility rate of ewes ovulating in response to the treatment was higher than 60% in all the groups (Table 2), with a trend of being higher in the GnRH-CIDR-GnRH group than in the CIDR-GnRH group (*p* = 0.06). Hence, finally, the fertility rate considering treated ewes was similar in the CIDR-eCG and GnRH-CIDR-GnRH groups and numerically higher in both groups than in the CIDR-GnRH group.

## 4. Discussion

The results of the present study indicate that the administration of GnRH after 5 days of CIDR-based protocols gives reproductive responses (in terms of ewes responding to the treatment with the appearance of estrus, preovulatory LH surge, and ovulation) that are similar to the classical protocols based on the use of eCG. Overall, the onset of these events was earlier in the group treated with eCG at CIDR removal (CIDR-eCG) than in the groups treated with GnRH at 56 h after CIDR removal (CIDR-GnRH and GnRH-CIDR-GnRH) and especially in the group treated with a first GnRH dose at CIDR insertion (GnRH-CIDR-GnRH). 

The administration of GnRH after CIDR withdrawal was performed at 56 h to mimic the timing of FTAI. Administration of GnRH at timing of FTAI, instead of 24–36 h after CIDR removal [13], diminishes the handling of the animals, which is always important in practice. However, under the conditions of the current study, there was no influence of the GnRH injection on the timing of onset of estrus behavior and even in the beginning of the preovulatory surge, which had occurred previously in all the animals. If using our proposed protocol for FTAI, administration of GnRH at 56 h would be important for avoiding animals with later preovulatory LH surges, which may negatively affect fertility, since GnRH treatment induces the LH surge within 1–4 h post-administration [20]. A possible advance of the timing of GnRH administration, weighing handling costs and the possible benefits of inducing an earlier and more synchronous LH peak, should be tested under field conditions since the design of the present study was mainly focused on studying the characteristics of estrus, preovulatory LH surge, and ovulation (which requires a high number of successive samples from a small number of animals) rather than the fertility yields (which requires a large number of animals).

The scenario in the CIDR-eCG and CIDR-GnRH groups, bearing in mind that the endogenous LH peak occurred prior to the GnRH injection, was therefore equivalent to our previous study comparing 5 days of CIDR treatment with or without eCG [4]. The comparison of results shows that differences in the timings of occurrence of estrus behavior and ovulation between animals with or without eCG are similar between the previous and the current study, confirming that the appearance of estrus is earlier when applying eCG. However, there are some differences in these features between studies, which may be related to the different time of the year since the occurrence of these events was earlier in the present trial (performed in midbreeding season, December) than in the previous one (performed in late-breeding season, April). These results suggest the need for further studies to determine the influence of seasonality but, at the same time, support the use of the GnRH injection at 56 h for counteracting possible delays in the endogenous LH surge, when eCG is not used, at the beginning or the end of the reproductive season. A further step would be to study the usefulness of this protocol for inducing synchronized and fertile ovulations during the anestrous season, which is the main limiting factor in case of eCG shortage, since gonadotrophin secretion and therefore ovulation are depressed in anestrous [21].

In the present trial, the timing of appearance of estrus, preovulatory LH surge, and ovulation after CIDR withdrawal were even more delayed after administration of the first GnRH dose at CIDR insertion (group GnRH-CIDR-GnRH). The differences were statistically significant when compared with the group treated with eCG, which may be an effect of the follicle-stimulating hormone (FSH) and LH activities of this hormone and the earlier timing of eCG administration, as well as numerically when compared with the CIDR-GnRH group. The delay in the preovulatory events found in the GnRH-CIDR-GnRH group is possibly related to the effect of the exogenous GnRH surge on follicular dynamics at the beginning of the follicular wave induced by the CIDR treatment. The CIDR insertion causes an abrupt increase in progesterone and a subsequent decrease in pituitary LH secretion, which induce atresia of all the large follicles present at the ovaries and the appearance of a new follicular wave [5]. The administration of GnRH causes the opposite effect, since it induces an endogenous LH surge, but has the same final result: the dominant follicle cannot ovulate and therefore undergoes luteinization, so a new follicular wave is initiated [10]. However, the GnRH injection not only acts on LH secretion but also on FSH secretion, which increases in response to GnRH stimulation in the absence of the negative feedback of estradiol and inhibin [22,23]. The best example is the FSH peak occurring immediately after the preovulatory LH peak and at about the time of ovulation, which is identified as being responsible for the development of the first follicular wave of the following estrous cycle [24]. In sheep, inhibin is secreted by follicles that are ≥3 mm in size [25,26], so we can hypothesize that the preovulatory follicle growing after GnRH synchronization of the wave would emerge from the pool of gonadotrophin-responsive follicles around 3 mm in size, which is the optimal follicular population for assisted reproductive techniques in sheep [27]. Hence, by injecting GnRH, we are inducing a similar scenario to Day 0 of the estrous cycle—the best scenario for applying assisted reproductive techniques (the so-called Day 0 Protocol in sheep MOET programs [28]). Afterwards, the CIDR assures high serum plasma progesterone concentrations which are necessary during follicular development to ensure adequate oocyte health at ovulation [29].

The differences among the GnRH-CIDR-GnRH group and the CIDR-GnRH and CIDR-eCG groups were mainly determined by the timing of onset of estrus behavior (i.e., the time needed by the preovulatory follicle to reach its maximal estradiol secretion and therefore to induce estrus signs), since timing and intervals of preovulatory LH surge and ovulation were similar among the three treatments. In fact, the ranges of appearance of preovulatory LH surge and ovulation were narrower in the GnRH-CIDR-GnRH group than in the CIDR-GnRH and CIDR-eCG groups, which reinforces evidence of a better synchronization of the follicular growth in such a group.

On the other hand, we have to highlight that the CIDR-eCG group had the widest range of occurrence of ovulations, from 52 to 76 h after CIDR withdrawal, after a wide range of occurrence of the preovulatory LH surge, from 28 to 56 h after CIDR withdrawal, and three peaks of onset of estrus behavior (with two groups of around 26%–27% of the sheep, each one having estrus signs at 24 and 44 h, and a third group of around 30% of the animals showing estrus behavior at 32–36 h after CIDR withdrawal). Such patterns of appearance of estrus, preovulatory LH surge, and ovulation suggest a heterogeneous follicle population stimulated to ovulate by the use of eCG. Hence, these results suggest that eCG may be the best option, and has been for around 70 years, but not a good one for managing the follicular phase after progesterone-based estrus synchronization, bearing in mind its long half-life and prolonged effect on follicular growth [30].

## 5. Conclusions

Protocols based on short-term (5 days) CIDR treatments and a double administration of GnRH (one at device insertion and one around the timing of fixed-time artificial insemination) assure the occurrence of fertile and synchronized ovulations for protocols based on the use of short-term CIDR treatments and eCG at device withdrawal.

## Figures and Tables

**Table 1:** Percentage and timing of occurrence (hours ± SEM) of estrus behavior, preovulatory luteinizing hormone (LH) surge, and ovulation in ewes treated with a single i.m. dose of 400 IU of eCG at CIDR removal (group CIDR-eCG), with a single dose of GnRH at 56 h after CIDR removal (group CIDR-GnRH), and with two GnRH doses—one at CIDR insertion and one 56 h after CIDR removal (group GnRH-CIDR-GnRH).

Event	CIDR-eCG (n = 19)	CIDR-GnRH (n = 19)	GnRH-CIDR-GnRH (n = 19)
Occurrence of estrus behavior (%)	17/19 (89.5)	17/19 (89.5)	16/19 (84.2)
Timing of estrus behavior after CIDR removal (range)	34.1 ± 2.0 ^a^ (24–44)	39.3 ± 2.0 ^b^ (28–52)	39.8 ± 2.2 ^b^ (24–52)
Occurrence of preovulatory LH surge (%)	17/17 (100)	17/17 (100)	16/16 (100)
Timing of preovulatory LH surge after CIDR removal (range)	42.2 ± 3.0 ^a^ (28–56)	44.4 ± 2.3 ^a,b^ (32–52)	50.7 ± 1.9 ^b^ (44–56)
Timing of preovulatory LH surge after onset of estrus behavior (range)	8.0 ± 1.0 (4–12)	6.7 ± 1.6 (4–16)	7.5 ± 1.6 (4–16)
Occurrence of ovulation (%)	17/17 (100)	17/17 (100)	16/16 (100)
Timing of ovulation after CIDR removal (range)	65.8 ± 2.3 ^a^ (52–76)	68.4 ± 2.5 ^a,b^ (60–80)	73.8 ± 2.1 ^b^ (68–84)
Timing of ovulation after onset of estrus behavior (range)	31.6 ± 0.8 (28–36)	30.7 ± 0.9 (28–36)	30.2 ± 1.0 (28–36)
Timing of ovulation after onset of preovulatory LH surge (range)	24.0 ± 1.1 (16–28)	24.0 ± 1.4 (16–28)	22.5 ± 1.3 (16–28)

Different superscripts indicate significant differences among treatments (a ≠ b: *p* < 0.05).

**Table 2:** Mean (± SEM) number of corpora lutea and plasma progesterone concentrations (ng/mL) and fertility rate in ewes treated with a single i.m. dose of 400 IU of eCG at CIDR removal (group CIDR-eCG), with a single dose of GnRH at 56 h after CIDR removal (group CIDR-GnRH), and with two GnRH doses—one at CIDR insertion and one 56 h after CIDR removal (group GnRH-CIDR-GnRH).

Parameter	CIDR-eCG	CIDR-GnRH	GnRH-CIDR-GnRH
Number of corpora lutea (range)	2.1 ± 0.2 ^a^ (1–4)	1.3 ± 0.2 ^b^ (1–2)	1.6 ± 0.2 ^a^ (1–2)
Plasma progesterone concentrations (range)	5.9 ± 1.0 (2.3–7.8)	5.1 ± 0.6 (2.9–7.1)	4.9 ± 0.7 (1.5–7.0)
Fertility rate with regards to ewes ovulating (%)	13/17 (76.5)	11/17 (64.7)	13/16 (81.3)
Fertility rate with regards to treated ewes (%)	13/19 (68.4)	11/19 (57.9)	13/19 (68.4)

Different superscripts indicate significant differences among treatments (a ≠ b: *p* < 0.05).
